# LRIG1 acts as a critical regulator of melanoma cell invasion, migration, and vasculogenic mimicry upon hypoxia by regulating EGFR/ERK-triggered epithelial–mesenchymal transition

**DOI:** 10.1042/BSR20181165

**Published:** 2019-01-08

**Authors:** Wei Li, Yubo Zhou

**Affiliations:** 1 Department of Burns, Sichuan Academy of Medical Sciences and Sichuan Provincial People’s Hospital, Chengdu 610072, P.R. China; 2 Department of Emergency, Sichuan Academy of Medical Sciences and Sichuan Provincial People’s Hospital, Chengdu 610072, P.R. China

**Keywords:** EGFR/ERK pathway, hypoxia, LRIG1, melanoma, metastasis, vasculogenic mimicry

## Abstract

Intratumoral hypoxia is a well-known feature of solid cancers and constitutes a major contributor to cancer metastasis and poor outcomes including melanoma. Leucine-rich repeats and Ig-like domains 1 (LRIG1) participate in the aggressive progression of several tumors, where its expression is frequently decreased. In the present study, hypoxia exposure aggravated melanoma cell invasion, migration, vasculogenic mimicry (VM), and epithelial–mesenchymal transition (EMT). During this process, LRIG1 expression was also decreased. Importantly, overexpression of LRIG1 notably counteracted hypoxia-induced invasion, migration, and VM, which was further augmented after LRIG1 inhibition. Mechanism analysis corroborated that LRIG1 elevation muted hypoxia-induced EMT by suppressing E-cadherin expression and increasing N-cadherin expression. Conversely, cessation of LRIG1 further potentiated hypoxia-triggered EMT. Additionally, hypoxia stimulation activated the epidermal growth factor receptor (EGFR)/ERK pathway, which was dampened by LRIG1 up-regulation but further activated by LRIG1 inhibition. More important, blocking this pathway with its antagonist erlotinib abrogated LRIG1 suppression-induced EMT, and subsequently cell invasion, migration, and VM of melanoma cells under hypoxia. Together, these findings suggest that LRIG1 overexpression can antagonize hypoxia-evoked aggressive metastatic phenotype by suppressing cell invasion, migration, and VM via regulating EGFR/ERK-mediated EMT process. Therefore, these findings may provide a promising target for melanoma therapy.

## Introduction

Melanoma is the most prevalent and aggressive form of skin cancer, with steadily increasing incidence and mortality worldwide [1]. Melanoma can be cured by resection in the early primary stage. However, despite treatment advances, the metastatic phenotype that develops in the late stages defies the current therapeutic modality. Survival beyond 10 years is poor and less than 10% for metastatic melanoma [2]. Therefore, it is urgent to explore the molecular mechanisms underlying metastatic progression of melanoma and identify effective therapeutic targets.

The hypoxic microenvironment is a proverbial feature of locally advanced solid tumors, including melanoma. Presence of hypoxia within tumor mass is associated with treatment failure and poor prognosis in patients [3,4]. Convincing evidence has supported hypoxia as a critical regulator in the initiation and development of solid cancers [5,6]. Tumor cells can adapt to hypoxia by altering their metabolism, which facilitates tumor metastasis by enhancing cancer cell invasion and migration [5,7]. Compelling research has identified hypoxia as a contributor to vasculogenic mimicry (VM) formation, a milestone event in tumorigenesis [8,9]. VM refers to the unique ability of aggressive cancer cells to form embryonic vasculogenic networks that are essential for metastatic capacity and growth in aggressive cancers under hypoxic conditions [10]. Intriguingly, hypoxia also induces epithelial–mesenchymal transition (EMT), a key process related to cancer cell invasion, migration, and VM [7]. Thus, targetting tumor hypoxia has been validated as a promising therapeutic strategy against cancer progression including metastasis [11].

Leucine-rich repeats and Ig-like domains 1 (LRIG1), located at chromosomal band 3p14, is a type I transmembrane member of the LRIG family. Aberrant reduction in LRIG1 has been confirmed in several cancers [12,13]. LRIG1 also can serve as a potential prognostic marker in patients with a variety of tumors [14,15]. LRIG1 often functions as a suppressor in tumorigenesis by acting as a negative regulator of epidermal growth factor receptor (EGFR) signaling [16]. Down-regulation of LRIG1 aggravates the aggressive properties of glioma cells by activating the EGFR pathway [16]. While restoring LRIG1 expression reportedly inhibits bladder cancer cell growth by suppressing EGFR activity [17]. Moreover, LRIG1 loss increases the risk of early and late relapse of breast cancer [13], but its overexpression inhibits EMT and cell invasion [12]. Emerging evidence has substantiated that low expression of LRIG1 correlates with poor survival in patients with melanoma [18]. Previous research corroborated the decrease in LRIG1 in glioblastoma cells upon hypoxia conditions, and its enhancement inhibited tumor growth and cell migration [19]. Unfortunately, the role of LRIG1 in melanoma remains poorly defined.

The present study thus aimed to explore the role of LRIG1 in hypoxia-induced aggressive invasion, migration, and VM in melanoma cells. Furthermore, the underlying molecular mechanism was also elucidated.

## Materials and methods

### Antibodies

Rabbit against human EGFR (#4267), p-EGFR (#3777), ERK (#4695), p-ERK (#9101) were purchased from Cell Signaling (Danvers, MA, U.S.A.). The antibodies against human LRIG1 (ab36707), vascular endothelial (VE)-cadherin (ab33168), E-cadherin (ab40772), and vimentin (ab8978) were obtained from Abcam (Cambridge, U.K., U.S.A.).

### Cell culture

The human melanoma cell line A2058 was bought from the American Type Culture Collection (ATCC, Manassas, VA, U.S.A.). All cells were grown in Dulbecco’s modified Eagle’s medium (DMEM) including 10% FBS and the antibiotics penicillin (100 µg/ml) and streptomycin (100 µg/ml). For normal incubation, all cells were housed in a humidified incubator containing 5% CO_2_ and 95% air (normoxia) at 37°C.

### Hypoxia exposure

For hypoxia exposure, cells were incubated in DMEM, and maintained in the incubator flushed with a gas mixture with 5% CO_2_, 94% N_2_, and 1% O_2_. Cells were then collected at 4, 12, 24, and 48 h post incubation.

### Quantitative real-time PCR

Total RNA from cells was extracted with TRIzol reagent (TaKaRa, Dalian China). The first-strand cDNA was then synthesized from 5 μg of total RNA using the SuperScript III First-strand Synthesis System (Invitrogen, Carisbad, CA, U.S.A.). To quantitate the transcript levels of LRIG1, real-time PCR was performed using the SYBR Premix Ex Taq™ II Kit (Takara) with the specific primers for LRIG1 (5′-GGCCTACCTTTCCTTAGAAGTG-3′, sense; 5′-GCCAGGTTGAGCTCCTTTAT-3′, antisense). For normalization, β-actin was introduced as a quality control to normalize gene expression. The primers for β-actin were 5′-GTTGCCCTGAGGCTCTTT-3′ (sense) and 5′-GATGTCCACGTCACACTTCA-3′ (antisense). The quantitative real-time PCR (qRT-PCR) was performed on an Applied Biosystems 7900HT (Foster City, CA, U.S.A.). The relative expression of LRIG1 was calculated using the 2^−ΔΔ*C*^_t_ method.

### Construction of recombinant LRIG1 vector and transfection

The full-length cDNA of LRIG1 was amplified by PCR. Following digestion with the restriction enzymes of *BamH* I and *Xho* I, the obtained sequences were inserted to the *BamH* I and *Xho* I cloning sites of the pCDNA3.1(+) construct (Invitrogen) to prepare the recombinant pcDNA3.1-LRIG1 plasmid. When grown up to 70% confluence, cells were transfected with the recombinant vector (15 µg) using Lipofectamine 2000 (Invitrogen). Cells that were transfected with empty vector were defined as the negative control. Twenty-four hours later, the transfection efficiency was evaluated by Western blotting.

### Knockdown of LRIG1 by siRNA transfection

To silence the expression of LRIG1 in A2058 melanoma cells, the scramble siRNA (5′-ACTACCGTTGTTATAGGTG-3′) (si-NC) and siRNA sequence targetting LRIG1 (5′-GCTCAGAACTCAGCCGGTTCTATTT-3′) were synthesized by Invitrogen. For siRNA transfection, 100 nmol/l of si-LRIG1 or si-NC was transfected into cells with the help of Lipofectamine 2000 according to the manufacturer’s instructions. The subsequent effect of siRNA transfection was assessed by Western blotting.

### Western blotting analysis

Cells under various treatments were incubated with radio-immunoprecipitation (RIPA) lysis buffer to prepare the protein lysates. Following centrifugation, protein contents were detected using a BCA kit (Pierce, Rockford, IL, U.S.A.). Afterward, equal concentration of protein was separated by 12% SDS/PAGE, and then transferred on to the PVDF membrane. The membrane was subsequently incubated with 5% non-fat milk to interdict the non-specific binding. Then, the primary antibodies against human LRIG1, EGFR, p-EGFR, ERK, p-ERK, VE-cadherin, E-cadherin, and vimentin were added for further incubation at 4°C. The membrane was then incubated with horseradish peroxidase-linked secondary antibodies for 2 h. After washing with Tris-buffered saline with Tween (TBST), immunoreactive bands were visualized by the ECL reagent (Beyotime, Shanghai, China). The band intensities were quantitated using a Quantity One software (Bio-Rad, U.S.A.).

### Transwell invasion and migration assay

Cells were treated with LRIG1 vector, si-LRIG1 or EGFR pathway inhibitor erlotinib (0.1 μM for 2 h), prior to exposure to hypoxia condition. For cell invasion assay, the transwell chambers containing 8-µm pore size inserts were pre-coated with Matrigel (1:8 diluted in culture medium) (BD Bioscience, San Jose, CA, U.S.A.). Then, cells (1 × 10^5^) were resuspended in serum-free DMEM and added to the upper chamber. The lower chamber was replenished with the medium containing 10% FBS. After culturing for 24 h, the upper chamber was wiped out with a cotton swab to clear the residual cells. The same protocols were performed to evaluate cell migration ability just without Matrigel coat. The invasive and migratory cells were ultimately stained with 0.1% Crystal Violet and photographed under a light microscope (×100 magnification). The number of cells was counted from five randomly selected visual fields.

### Detection of VM formation by 3D cultures

To evaluate A2058 melanoma cell VM formation *in vitro*, a 3D Matrigel culture was used. Briefly, the 24-well plates were pre-coated with Matrigel (0.1 ml/well) and used for the subsequent experiments when the Matrigel was solidified. After transfection with LRIG1 plasmids or vector, cells were trypsinized and suspended in DMEM. Then, cells (2 × 10^5^/well) were plated and incubated on the surface of Matrigel for 24 h at 37°C under hypoxic or normoxic environments. The formation of VM was visualized under a light microscope and presented as the percentage of the control group.

### Statistical analysis

All observations were validated by at least three independent experiments. All data were analyzed by a SPSS 16.0 software package (Chicago, IL, U.S.A.) and presented as the mean ± S.D. For statistical analysis, the *t* test was used for comparisons between two groups, and ANOVA for three or more groups, followed by Student–Newman–Keuls (SNK) post hoc test.

## Results

### Hypoxia evokes a more aggressive phenotype in melanoma cells

**Figure 1:**
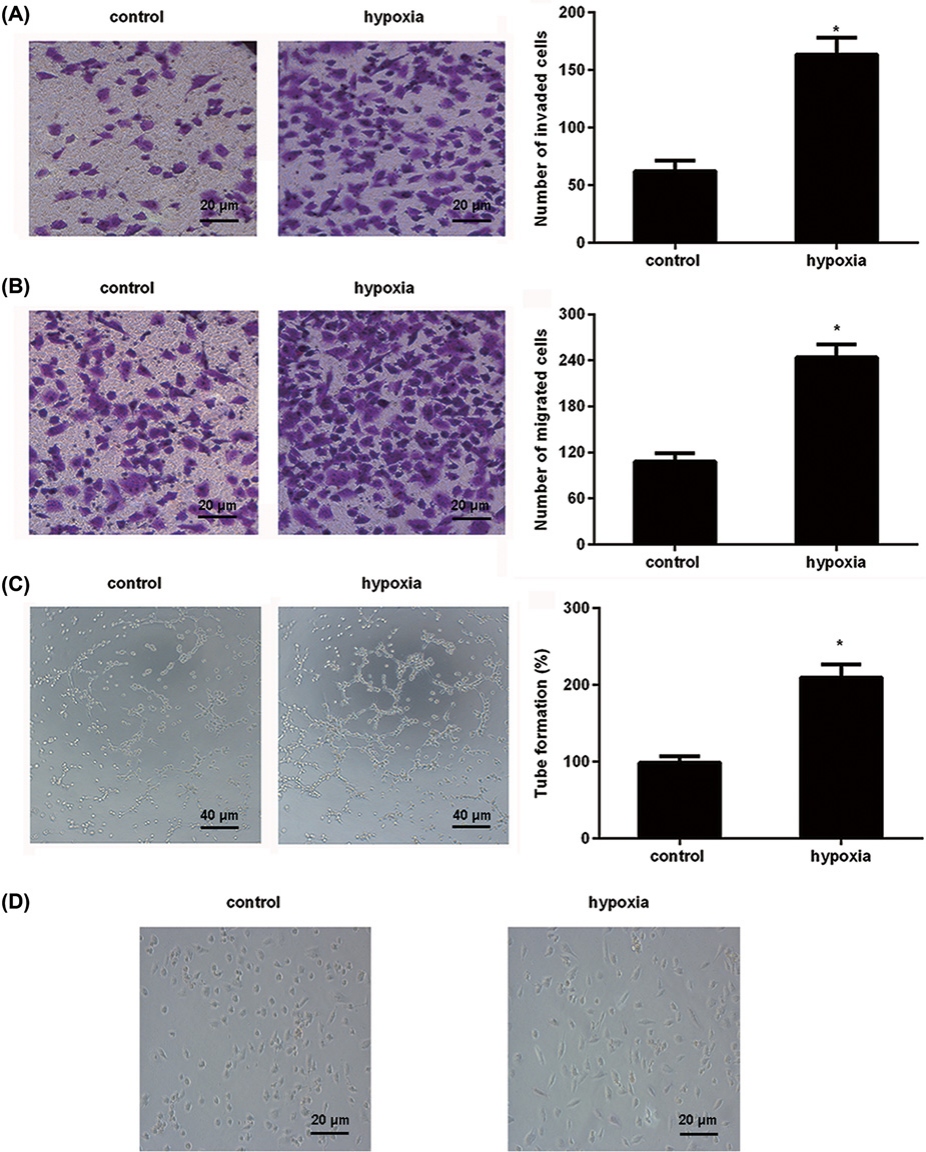
Hypoxia induced more aggressive phenotype in melanoma cells (**A**) Cells were exposed to normoxia or hypoxia for 12 h. Then, cell invasion ability was evaluated by Transwell analysis (×200 magnification). (**B**) Transwell assay was performed to assess cell migration ability (×200 magnification). (**C**) Cells were subjected to 3D Matrigel culture for 24 h under hypoxia exposure. Then, the formation of tube-like structure was photographed under light microscope (×100 magnification). (**D**) Morphological changes were observed under light microscope (×200 magnification). *P<0.05 compared with normoxia group.

Hypoxia is the characteristic of solid tumors, including melanoma. After exposure to hypoxia, the invasion ability of melanoma cells was increased (Figure 1A). Moreover, hypoxia treatment also promoted cell migration (Figure 1B). Simultaneously, exposure to hypoxia induced the formation of VM (Figure 1C). Additionally, cells upon hypoxia condition underwent typical morphological changes of EMT from epithelial morphology to spindle-like phenotype (Figure 1D). These results indicate that hypoxia induces an aggressive phenotype in melanoma cells.

### Expression of LRIG1 is inhibited in melanoma cells exposed to hypoxia

**Figure 2:**
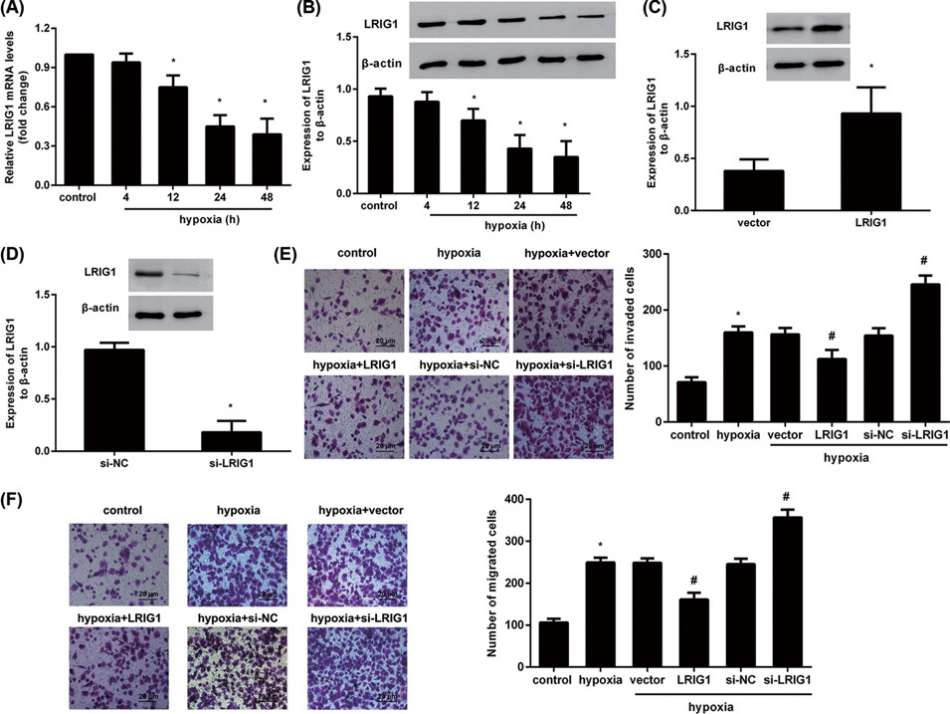
Effects of LRIG1 expression on hypoxia-augmented melanoma cell invasion and migration (**A**) After exposure to hypoxia for 4, 12, 24, and 48 h, the mRNA levels of LRIG1 were detected by qRT-PCR. (**B**) The protein levels of LRIG1 at 24 h post incubation were analyzed by Western blotting. *P<0.05 compared with normoxia control group. (**C**) Cells were transfected with the recombinant plasmid of LRIG1 or empty vector, and then LRIG1 expression was determined. *P<0.05 compared with vector group. (**D**) After transfection with LRIG1 siRNA or si-NC, the protein expression of LRIG1 was evaluated. *P<0.05 compared with si-NC group. (**E**,**F**) Effects of LRIG1 overexpression or inhibition on hypoxia-induced melanoma cell invasion (E) and migration (F). *P<0.05 compared with control group. ^#^*P*<0.05 compared with normoxia group.

To clarify the role of LRIG1 in hypoxia-aggravated melanoma progression, the expression of LRIG1 was explored. As shown in Figure 2A, the transcript levels of LRIG1 was gradually reduced with increasing hypoxia exposure, relative to the normoxia control group. No statistical differences in the *LRIG1* mRNA transcript were observed in cells exposed to hypoxia for 24 and 48 h. Furthermore, in contrast with the control group, exposure to hypoxia for 24 h evoked a 0.43-fold decrease in protein expression of LRIG1 (Figure 2B).

### Elevation of LRIG1 antagonizes hypoxia-induced metastatic potential of melanoma cells, but is aggravated after LRIG1 cessation

To explore the role of LRIG1 in the development of melanoma responding to hypoxia environment, we investigated the effects of LRIG1 overexpression or silencing on the metastatic potential of melanoma cells. As shown in Figure 2C, transfection with the recombinant LRIG1 plasmids enhanced LRIG1 expression in melanoma A2058 cells. In contrast, the protein expression of LRIG1 was decreased after si-LRIG1 transfection (Figure 2D). Further functional analysis corroborated that hypoxia-induced cell invasion was abrogated following LRIG1 overexpression (Figure 2E). Conversely, LRIG1 cessation further promoted hypoxia-triggered invasion (Figure 2E). Analogously, the increase in cell migration was restrained in LRIG1 overexpression group, but was further enhanced in LRIG1 silencing group (Figure 2F).

### LRIG1 overexpression weakens hypoxia-enhanced VM and EMT, and its inhibition elevates hypoxia-enhanced VM and EMT

**Figure 3:**
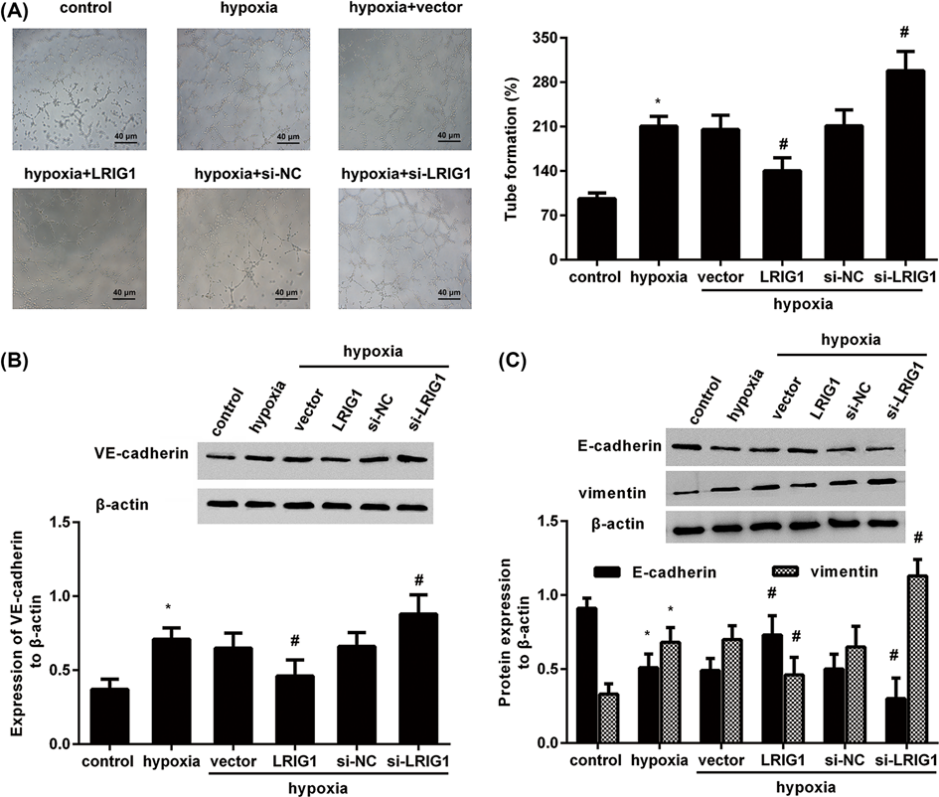
LRIG1 elevation weakens, but its inhibition increased hypoxia-enhanced VM and EMT (**A**) After transfection with LRIG1 plasmids or si-LRIG1, cells were then subjected to 3D Matrigel culture under hypoxia conditions for 24 h. Then, the formation of VM was observed under a light microscope. (**B**) The expression of VM-related marker VE-cadherin was detected by Western blotting. (**C**) The expression of EMT markers E-cadherin and vimentin was measured by Western blotting. *P<0.05 compared with control group. ^#^*P*<0.05 compared with normoxia group.

Increasing evidence has corroborated that aggressive carcinoma cells can acquire the unique ability to form VM, which is strongly related to metastatic potential and poor prognosis in cancers, including melanoma [9,10]. We therefore further elucidated the function of LRIG1 in hypoxia-induced VM of melanoma cells by establishing a 3D culture model. Interestingly, hypoxia exposure potentiated VM formation, which was counteracted by LRIG1 overexpression (Figure 3A). However, the formation of channel-like structures under hypoxia was further augmented after LRIG1 suppression (Figure 3A). Simultaneously, expression of VE-cadherin, a representative marker relative to VM, also was increased after LRIG1 inhibition, but was dampened following LRIG1 overexpression (Figure 3B). Additionally, elevation of LRIG1 neutralized hypoxia-induced EMT by restoring hypoxia-evoked epithelial marker E-cadherin decrease and inhibiting hypoxia-increased mesenchymal marker vimentin expression (Figure 3C,D). However, suppression of LRIG1 reinforced hypoxia-triggered E-cadherin down-regulation and vimentin up-regulation.

### Effects of LRIG1 on the activation of the EGFR/ERK pathway in response to hypoxia

**Figure 4:**
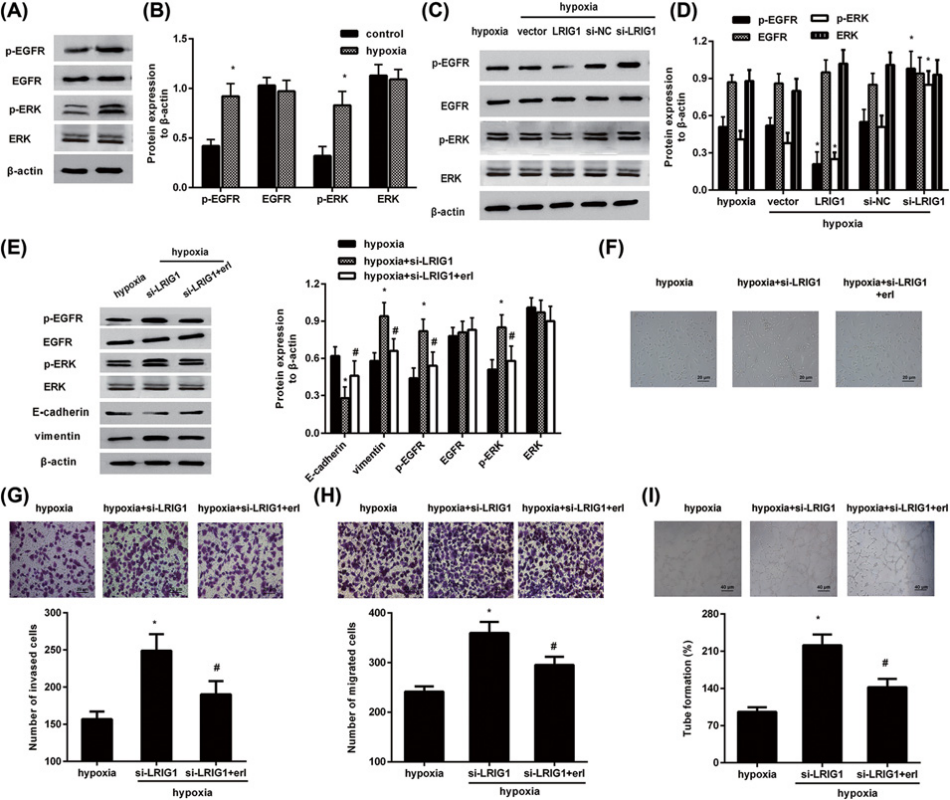
Effects of LRIG1 on hypoxia-induced activation of the EGFR/ERK pathway (**A**,**B**) After exposure to hypoxia for 24 h, the expression of p-EGFR, EGFR, p-ERK, and ERK was detected. *P<0.05 compared with control group. (**C**,**D**) Cells were transfected with the LRIG1 vector or si-LRIG1, prior to hypoxia exposure. Then, the activation of the EGFR/ERK pathway was evaluated. *P<0.05 compared with hypoxia group. (**E**) Following pretreatment with erlotinib for 2 h, the expression of E-cadherin, vimentin, and the activation of the EGFR-ERK pathway in LRIG1-silenced cells upon hypoxia was measured. (**F**) The effects on morphological changes were assessed under light microscope (×200 magnification). (**G**–**I**) The subsequent effects on cell invasion (G), migration (H), and VM formation (I) were also evaluated. *P<0.05 compared with hypoxia group. ^#^*P*<0.05 compared with si-LRIG1+hypoxia group.

Aberrant activation of the EGFR/ERK pathway has been confirmed in various carcinomas and is relative to cancer development [20,21]. Western blotting analysis confirmed considerable elevation of p-EGFR expression after hypoxia exposure, concomitant with the subsequent increase in p-ERK protein levels (Figure 4A,B). To clarify the mechanism underlying the roles of LRIG1 in hypoxia-induced aggressive potential of melanoma cells, we explored the effects of LRIG1 on EGFR/ERK signaling. As shown in Figure 4C,D, overexpression of LRIG1 notably reduced the expression of p-EGFR and p-ERK induced by hypoxia, indicating that LRIG1 elevation muted hypoxia-evoked activation of the EGFR/ERK pathway. However, depression of LRIG1 further activated the EGFR/ERK signaling upon hypoxia stimulation (Figure 4C,D).

### Abrogation of the EGFR/ERK pathway by erlotinib counteracts LRIG1 cessation-induced aggressive metastasis potential in melanoma cell under hypoxia

To further substantiate whether LRIG1 regulates hypoxia-induced invasion, migration, and VM via the EGFR/ERK pathway, erlotinib, the highly selective antagonist of EGFR, was used to inhibit EGFR signaling. As shown in Figure 4E, preconditioning with erlotinib blocked LRIG1 downregulation-induced activation of the EGFR/ERK pathway. Additionally, erlotinib also antagonized LRIG1 knockdown-induced decrease in E-cadherin and increase in vimentin in hypoxia-stimulated melanoma cells, indicating that LRIG1 depression could enhance hypoxia-induced EMT by activating the EGFR/ERK pathway. Furthermore, cessation of LRIG1 further induced EMT under hypoxia conditions, which was reversed following erlotinib pretreatment (Figure 4F). Concomitantly, the increased invasiveness of hypoxic cells after LRIG1 cessation also reverted after erlotinib treatment (Figure 4G). Furthermore, hypoxia-induced cell migration was further augmented after LRIG1 knockdown, which was counteracted following erlotinib stimulation (Figure 4H). LRIG1 inhibition further enhanced hypoxia-induced VM, which was attenuated when erlotinib blocked the EGFR signaling (Figure 4I). Together, the EGFR/ERK pathway may be responsible for LRIG1-mediated melanoma cell aggressive metastasis upon hypoxia by regulating cell invasion, migration, and VM via EMT.

## Discussion

Tumor hypoxia is a well-known phenomenon in most solid cancers, including melanoma. Accumulating evidence has confirmed the correlation between hypoxia and poor prognosis [3]. During intratumoral hypoxia, cancer cells can adapt to hypoxia and promote invasion and migration. Analogous with previous research [22], hypoxia exposure induced cell invasion and migration. Furthermore, EMT change was also observed after hypoxia exposure, which is similar to former findings in colorectal cancer [23]. Notably, hypoxia also induced VM in melanoma cells. Under hypoxia conditions, tumor cells form vasculature to supply nourishment for tumor growth and metastasis [10]. Therefore, these findings suggest that hypoxia aggravates the metastatic potential of melanoma by inducing cell invasion, migration, VM, and EMT.

We next investigated the mechanism underlying hypoxia-induced aggressive potential of melanoma cells, and confirmed a dramatic down-regulation of LRIG1 in melanoma cells upon hypoxia. Analogous with our study, down-regulation of LRGI1 under hypoxia also has been detected in glioblastoma cells [19]. LRIG1 is frequently decreased and often correlated with poor outcomes in various cancers, including melanoma [18]. In glioblastoma, LRIG1 silencing promotes cell proliferation and invasion [16]. LRIG1 also inhibits EMT and invasion of basal-like breast cancer cell [12]. These findings promote us to explore whether LRIG1 exerts the crucial role in hypoxia-induced melanoma progression. As expected, LRIG1 elevation antagonized hypoxia-induced cell invasion, migration, and VM, which were further aggravated after LRIG1 cessation. Analogously, overexpression of LRIG1 also inhibited hypoxia-induced VM in glioma cells [24]. These data suggest that LRIG1 may act as a pivotal participator in hypoxia-evoked aggressive progression of melanoma.

Existence of EMT, which is the key event in cancer metastasis, has been widely validated in aggressive carcinomas. EMT is a cellular process characterized by the loss of the epithelial polarized cell junction and the gain of motile mesenchymal cells. Abundant evidence has validated that EMT is critical to cancer cell metastasis by enhancing cell invasion and migration [7,25]. For instance, enhancing hypoxia-induced EMT by long non-coding RNA (lncRNA) NORAD (non-coding RNA-activated by DNA damage) promoted pancreatic cancer cell invasion, migration, and tumor metastasis [7]. Cells that acquire EMT are more easier to form VM-like vascular channels. Previous research confirmed hypoxia-induced VM in ovarian carcinoma by inducing EMT [8]. To elucidate how LRIG1 involved in hypoxia-triggered metastatic potential of melanoma cells, we explored the effects of LRIG1 on EMT. According to our hypothesis, hypoxia exposure induced EMT, accompanied by a decrease in E-cadherin expression and increase in vimentin expression. However, LRIG1 overexpression counteracted hypoxia-triggered EMT, which was further augmented following LRIG1 suppression. So, the current data indicate that LRIG1 may account for hypoxia-induced metastasis of melanoma by enhancing cell invasion, migration, and VM via EMT.

EGFR signaling is a common pathway during carcinogenesis, and is frequently amplified in several cancers [17,20,21]. Blocking this pathway suppresses lung cancer growth and metastasis [21]. However, activating the EGFR and downstream ERK pathway by pinin facilitates cell proliferation, invasion, tumorigenic growth, and metastasis in colorectal cancer [20]. Intriguingly, the present study confirmed that hypoxia exposure activated the EGFR-ERK pathway. Similarly, the EGFR signaling also is activated by hypoxia in gastric cancer and glioma [24,26]. More important, overexpression of LRIG1 muted hypoxia-induced activation of the EGFR pathway, which was further activated by LRIG1 cessation. Blocking this pathway with its inhibitor, erlotinib attenuated LRIG1 inhibition-enhanced EMT and subsequent cell invasion, migration, and VM upon hypoxia. Similarly, overexpression of LRIG1 also abrogated hypoxia-induced VM by blocking EGFR signaling [24]. Thus, these results imply that the EGFR/ERK pathway is responsible for hypoxia-induced aggressive metastatic potential of melanoma cells.

Collectively, the present study first confirmed down-regulation of LRIG1 in hypoxia-treated melanoma cells. Intriguingly, elevation of LRIG1 counteracted hypoxia-enhanced metastatic potential by attenuating cell invasion, migration, and VM, which was further aggravated after LRIG1 cessation. Moreover, the EGFR/ERK pathway-mediated EMT was accounted for above process. Consequently, these findings corroborate how LRIG1 deteriorates hypoxia-triggered cancer metastasis in solid tumors, supporting a promising therapeutic target for melanoma intervention treatment.

## Abbreviations

DMEM: Dulbecco’s modified Eagle’s medium
EGFR: epidermal growth factor receptor
EMT: epithelial–mesenchymal transition
LncRNA: long non-coding RNA
LRIG1: leucine-rich repeats and Ig-like domains 1
NORAD: non-coding RNA-activated by DNA damage
qRT-PCR: quantitative real-time PCR
RIPA: radio-immunoprecipitation
TBST: Tris-buffered saline with Tween
VE-cadherin: vascular endothelial (VE)-cadherin
VM: vasculogenic mimicry
